# First microscopic and molecular parasitological survey of *Strongylus vulgaris* in Brazilian ponies

**DOI:** 10.1590/S1984-29612023036

**Published:** 2023-06-23

**Authors:** André Vianna Martins, Aline de Lima Coelho, Laís Lisboa Corrêa, Mariana Santos Ribeiro, Lucas Fernandes Lobão, João Pedro Siqueira Palmer, Lucas Cavalcante de Moura, Marcelo Beltrão Molento, Alynne da Silva Barbosa

**Affiliations:** 1 Laboratório de Bioagentes Ambientais, Departamento de Microbiologia e Parasitologia, Instituto Biomédico, Universidade Federal Fluminense – UFF, Niterói, RJ, Brasil; 2 Laboratório de Parasitologia e Doenças Parasitárias, Faculdade de Medicina Veterinária, Centro Universitário Serra dos Órgãos, Teresópolis, RJ, Brasil; 3 Laboratório de Parasitologia Clínica Veterinária, Universidade Federal do Paraná – UFPR, Curitiba, PR, Brasil; 4 Laboratório de Toxoplasmose e Outras Protozooses, Instituto Oswaldo Cruz, Fundação Oswaldo Cruz – Fiocruz, Rio de Janeiro, RJ, Brasil

**Keywords:** Brazilian pony, molecular diagnosis, large strongylids, intestinal parasites, Strongylus vulgaris, Pônei brasileiro, diagnóstico molecular, grande estrôngilo, parasito intestinal, Strongylus vulgaris

## Abstract

The frequency of gastrointestinal parasites with an emphasis on *Strongylus vulgaris* was investigated among the Brazilian Pony breed kept on farms in the municipality of Teresópolis, state of Rio de Janeiro. Fecal samples were collected in three stud farms: A (n= 22 animals), B (n= 3), and C (n= 2). Fecal samples were subjected to the quantitative Mini-FLOTAC technique, using three different solutions, and to qualitative techniques. The parasite prevalence was found to be 81.4%. Eggs from strongylids were identified in 74% of the ponies. Eggs of *Parascaris* spp. were detected in 22.7% of the animals, which were all females of farm A. At this locality, mares were kept with their foals in fenced paddocks all the time. The NaCl solution of d = 1.200 g/ml was generally the one that presented the highest frequency of diagnosis of nematode eggs and the highest mean of fecal eggs per gram. The fecal samples were also subjected to the polymerase chain reaction for amplification of DNA from the ITS2 region for *Strongylus vulgaris*. Twelve samples presented nucleotide sequences for *S. vulgaris*. In the end, this study revealed the high frequency (96.3%) of *S. vulgaris* among ponies on farms in Teresópolis, Rio de Janeiro, Brazil.

## Resumo

A frequência de parasitos gastrointestinais, com ênfase na pesquisa de *Strongylus vulgaris*, foi investigada entre os Pôneis Brasileiros criados em haras na cidade de Teresópolis, no estado do Rio de Janeiro. Amostras fecais foram coletadas em três haras: A (n=22 animais), B (n=3) e C (n=2). Amostras fecais foram submetidas à técnica quantitativa de Mini-FLOTAC utilizando três diferentes soluções e técnicas qualitativas. A prevalência de parasitos foi de 81,4%. Ovos de estrôngilos foram identificados em 74% dos pôneis. Ovos de *Parascaris* spp. foram detectados em 22,7% dos animais, sendo todos fêmeas do haras A. Nesta propriedade, as éguas eram mantidas com os pôneis em piquetes cercados durante todo o tempo. A solução de NaCl, com densidade de 1.200 g/ml, foi a que apresentou a maior frequência diagnóstica de ovos de nematoides e a maior contagem de ovos por grama de fezes. As amostras também foram submetidas à reação de polimerase em cadeia para amplificar DNA da região ITS2 de *Strongylus vulgaris*. Doze amostras fecais apresentaram sequências nucleotídicas de *S. vulgaris*. Ao final, este estudo demonstrou a alta frequência (96.3%) de *S. vulgaris* em pôneis mantidos em haras na cidade de Teresópolis, Rio de Janeiro, Brazil.

## Introduction

Ponies are present around the world and the Brazilian Association of Pony Horse-Breeders (ABCC Pônei) considers that there are two genuinely Brazilian breeds: the Brazilian Pony and the Piquira. The Brazilian Pony breed descended from the Shetland breed of Scotland and the Falabella of Argentina, together with some influence from animals coming from Paraguay and Uruguay (ABCC Pônei, 2022). ​Among the breed standards that have been selected, the height of the Brazilian Pony must not exceed 100 cm for males and 110 cm for females. This very small horse exhibits refined forms, as is proper for all dual-purpose horses, i.e., riding and light haulage (ABCC Pônei, 2022). Even though these animals are found in many farms in Brazil, where they are used mainly for leisure and equestrian events, there are still no consistent studies about the occurrence of gastrointestinal parasites and their impact to the animals.

It is known that horses can be infected by different nematodes, including strongylids, *Parascaris* spp., *Strongyloides westeri,* and *Oxyuris equi*, and by cestodes of the Anoplocephalidae family (Barriga, 1995; Molento, 2005). Among these, strongylids of the Strongylidae family, such as the subfamily Strongylinae (large strongylids), and the subfamily Cyathostominae (small strongylids; also known as cyathostomins) can be highlighted due to their high prevalence, pathogenicity and resistance to anthelmintics (Flanagan et al., 2013; Canever et al., 2013; Villa-Mancera et al., 2021). These parasites can cause severe gastrointestinal alterations, particularly diarrhea and considerable abdominal pain (Villa-Mancera et al., 2021; Molento & Vilela, 2021).

Given the importance of parasites to the health of the animals and the lack of information about the presence of these agents in ponies in Brazil, this study had the aim to determine the frequency of gastrointestinal helminths in the feces of the Brazilian Pony. Molecular diagnoses for *S. vulgaris* were made, as well as the estimate for helminth egg counts per gram (EPG) of feces with different flotation solutions using the Mini-FLOTAC chamber (Castro et al., 2017).

## Material and Methods

### Collection site for samples and data

Fecal samples were collected between August 2019 and July 2021, at three breeding farms located in the municipality of Teresópolis (latitude: 22° 24' 44” south; longitude: 42° 57' 59” west), in the state of Rio de Janeiro, southeast Brazil. The city is at an altitude of 871 meters above sea level under the Cfb climate zone (temperate with hot summers) according to the Köppen classification (Alvares et al., 2013). The city has a mean annual temperature of 18.5 ºC with an annual rainfall of 2080 mm^3^.

Three stud farms (A, B, and C) on which the Brazilian Pony breed was reared were included in the study. All of the ponies on these farms were housed in individual or collective stalls, always with access to paddocks. The stalls were made of masonry with a cement floor covered with wood shavings. On-farm A, there were paddocks with bare earth and little pasture material, while on farms B and C the paddocks were covered with grass. In the paddocks of all three farms, there were food and water at ground level. All the animals were fed with fiber-rich feed, preferentially consisting of chopped elephant grass (*Pennisetum purpureum*) and/or alfalfa hay (*Medicago sativa*), together with 1.0 kg of premix concentrate twice daily. In addition, the ponies received mineral salt and water *ad libitum*. There was generally one stallion pony on the farms, which performed natural mounting on the mares at specific times. The ponies receive anti-rabies vaccines annually and anti-influenza vaccines every six months.

### Collection of fecal samples, data recovery, and sampling

In total, 27 fecal samples were collected from the ponies: 22 on farm A (17 females and 8 males), 2 ponies on farm B (1 female and 1 male), and 3 ponies on farm C (1 female and 2 males). The age of the animals ranged from 6 months to 23 years. In all, 11 ponies were less than 3 years old, of which 10 belonged to farm A and 1 to farm C. As for the 16 animals older than or equal to 3 years old, 12 were from farm A, 2 were from farm B, and 2 were from farm C.

In addition to the fecal samples, information was obtained regarding the sex and age of the ponies, the farm management, the hygiene of the installations, and the regimens and procedures used for supplying anthelmintics. This information was obtained from each of the farms. During the sample collection, none of the ponies presented any clinical abnormalities and there were no histories of mortality on any of the farms. The animals were receiving monthly veterinary care, but no coproparasitological diagnoses were being made.

The fecal samples were collected directly from the rectum of the animals, and sent in isothermal boxes for processing in the laboratory. All the ponies had gone two months without receiving any anthelmintic treatment.

### Macroscopic analysis and parasitological techniques

The fecal samples were subjected to the Mini-FLOTAC technique, in which three floatation solutions were tested, as described by Cringoli et al. (2017): (1) sodium chloride (NaCl) with d = 1.200 g/ml; (2) zinc sulfate (ZnSO_4_) with d = 1.200 g/ml; and (3) ZnSO_4_ with d = 1.350 g/ml. After homogenization of the material, the fecal solution was applied to the Mini-FLOTAC chamber for observation (x10 and x40) using an Olympus BX 41 microscope (Tokyo, Japan). The helminth eggs were individually counted and identified. At the end of the counting, the value obtained was multiplied by the correction factor of 5. The centrifugation-flotation technique of Sheather (1923) was performed, as modified by Huber et al. (2003), and also the spontaneous sedimentation technique of Lutz (1919). The data from the animals, their management, and fecal results are presented according to the parasite positivity rate. The relevance of variables that had more than one category was evaluated, for each taxon of the parasite with the Fisher's Exact statistical test at a significance level of 5% using the Epi-Info software.

### DNA extraction, conventional PCR, and sequencing for *Strongylus vulgaris*

The process of DNA extraction from the samples was performed using 0.2 ml of the feces collected, which had previously been stored frozen in microtubes. The fecal material was subjected to DNA extraction using the QIAamp Fast DNA Stool Mini Kit (Qiagen, Hilden, Germany), following the manufacturer’s recommendations with modifications. Three cycles of thermal shock and an incubation stage in a thermoblock at 65 ºC with proteinase lysis buffer for 5 h were added. After extraction, the DNA was kept in a freezer until the polymerase chain reaction (PCR).

The DNA was subjected to PCR using the Invitrogen master mix Platinum Hot Start (Itapevi, Brazil) with the forward primer Sv-f (5′-GTATACATTAAATAGTGTCCCCCATTCTAG-3′) and the reverse primer Sv-r (5′-GCAAATATCATTAGATTTGATTCTTCCG-3′). The primers amplified an ITS2 region of the RNAr, described by Nielsen et al. (2008). Following this, a DNA fragment of approximately 169 bp was amplified by electrophoresis, which was compatible with *S. vulgaris* (Campbell et al., 1995; Hung et al., 1999). In the DNA extraction and in all the PCR runs, a negative control consisting of ultrapure water and a positive control from this laboratory, with the GenBank accession number OP550136, were inserted. In all, 5 µl of DNA extraction was used in the reaction. The amplified product was purified using the Invitrogen enzyme ExoSAP-it (Itapevi, Brazil) and sequenced using the technological platform network of Fundação Oswaldo Cruz (Fiocruz), in the city of Rio de Janeiro, Brazil.

The resultant nucleotide sequences were aligned and edited in the DNASTAR LaserGene Seqman software 7.1 (Madison, USA). Subsequently, the BLASTn analysis tool was used to compare the data obtained with reference sequences belonging to the same gene fragment stored in GenBank. The sequences were saved in Fasta mode and were aligned with other homologous sequences retrieved from GenBank, using the Mega X software. Phylogenetic inferences were made through maximum likelihood analyses for confirmation, using a bootstrap accessed with 1000 replications. The best evolutive model was selected based on the Akaike information criterion (AIC) using the W-IQ-Tree software (http://iqtree.cibiv.univie.ac.at/). The phylogenetic tree was edited and rooted using the Mega X software.

## Results

Out of the 27 fecal samples collected from the ponies, 22 (81.4%) were found to be positive for gastrointestinal parasites. Strongylid eggs were identified in 20 samples (74%), and *Parascaris* spp. was found in 5 samples (22.7%). Strongylid eggs were identified on all the stud farms, while *Parascaris* spp. eggs were only detected in fecal samples from animals on farm A (Table 1).

**Table 1:** Frequency (%), mean, and standard deviation for fecal egg count (EPG) of helminths using the Mini-FLOTAC in different solutions from ponies reared in Teresópolis, RJ, Brazil.

**Stud farm**	**Strongylids**	**EPG Mean and Standard deviation**			***Parascaris*spp.**	**EPG Mean and Standard deviation**			**Total**
		**ZnSO4 1.200**	**ZnSO4 1.350**	**NaCl 1.200**		**ZnSO4 1.200**	**ZnSO4 1.350**	**NaCl 1.200**	
**A (n= 22)**	16 (72.7%)	35.86 ± 167.75	25.12 ± 115.92	35.42 ± 153.33	5 (22.7%)	73.12 ± 271.34	48.69 ± 173.03	108.45 ± 392.06	18 (81.8%)
**B (n=3)**	3	30 ± 10	3.33 ± 5.77	40 ± 26.45	0	0	0	0	3
**C (n=2)**	1	7.5 ± 10.6	5 ± 7.07	10 ± 14.14	0	0	0	0	1
**Total (n= 27)**	20 (74%)				5 (18.5%)				22 (81.5%)

A, B, and C: stud farms where the Brazilian Pony breed was being reared.

Despite not having compared the efficiency of the solutions, the highest EPG means for strongylids was identified using Mini-FLOTAC with NaCl from farm B. As seen in diagnosing strongylids, the highest EPG value for *Parascaris* spp. was also obtained through NaCl (Table 1).

All the ponies presented EPG counts below 500, both for *Parascaris* spp. and for strongylids. The highest EPG values were 376 and 460 for strongylids. The highest number of positive samples was identified through Mini-FLOTAC using NaCl with d = 1.200 g/ml, for strongylids which were detected in 16 samples and *Parascaris* spp., in five.

Among the animals included in this study, 19 were female and eight were male; 11 were less than three years old and 16 had ages greater than or equal to three years. A greater percentage of fecal samples positive for intestinal nematodes came from male ponies. The frequency of positivity for these parasites was similar between the age categories in this study. Among the animals with fecal samples that were positive for *Parascaris* spp. we found in five females, and most of these animals were under three years of age (one pony was six months old and three were 1 year old). Although a higher parasite positivity was evidenced in younger females, no statistically significant relevance was observed for helminths from the same sex and age groups, nor any other information retrieved from the questionnaires (Table 2).

**Table 2:** Descriptive data relating to the frequency of positive samples for strongylids and *Parascaris* spp. according to information obtained through macroscopic examination of fecal samples and from the questionnaire applied to people in charge of the farms where the ponies were being reared, in Teresópolis, RJ, Brazil.

**Information from the macroscopic evaluation of feces and obtained from questionnaires**	**Strongylid** **(n= 17)**	***Parascaris* spp. (n= 3)**	**Strongylid and *Parascaris* spp. (n=3)**	**Total**
	**Positive (%)**	**Positive (%)**	**Positive (%)**	
**Consistency of fecal material**				
Solid (n=27)	17 (63%)	2 (7.4%)	3 (11.1%)	22 (81.5%)
* **Fecal staining**				
Greenish brown (n=3)	1 (33.3%)	0	2 (66.6%)	3 (100%)
Brown (n=24)	16 (66.6%)	2 (8.3%)	1 (4.1%)	19 (79.2%)
*Gender				
Female (n=19)	10 (52.6%)	2 (10.5%)	3 (15.8%)	15 (78.9%)
Male (n = 8)	7 (87.5%)	0	0	7 (87.5%)
*Age range				
< 3 years old (n=11)	5 (45.4%)	2 (18.2%)	2 (18.2%)	9 (81.8%)
≥ 3 years old (n=16)	12 (75%)	0	1 (6.3%)	13 (81.3%)
**Bed change**				
Monthly (n=27)	17 (63%)	2 (7.4%)	3 (11.1%)	22 (81.5%)
*Anthelmintic provided				
Ivermectin (n=5)	4 (80%)	0	0	4 (80%)
Ivermectin and praziquantel (n=22)	13 (59.1%)	2 (9.1%)	3 (13.6%)	18 (81.8%)
**Anthelmintic supply interval**				
Three-month intervals (n= 27)	17 (63%)	2 (7.4%)	3 (11.1%)	22 (81.5%)
**Dosages calculated of the anthelmintic**				
Estimate of each animal’s live weight (n=27)	17 (63%)	2 (7.4%)	3 (11.1%)	22 (81.5%)
*Water provided for animals				
Spring water (n=22)	13 (59.1%)	2 (9.1%)	3 (13.6%)	18 (81.8%)
Spring water with treatment (n=5)	4 (80%)	0	0	4 (80%)

* Variables analyzed by Fisher's Exact Test. All presented values ​​of p>0.05, not being statistically significant.

Regarding health management, all of the people in charge of the farms reported that the stall bedding material was changed monthly and that the ponies received spring water. However, on farms B and C, which accounted for five of the animals sampled, the spring water was subjected to prior treatment before being offered to the ponies. Regarding the anthelmintics supplied to the ponies, these consisted mainly of ivermectin, administered at three-month intervals at dosages calculated from the animal’s weight (Table 2).

**Figure 1:**
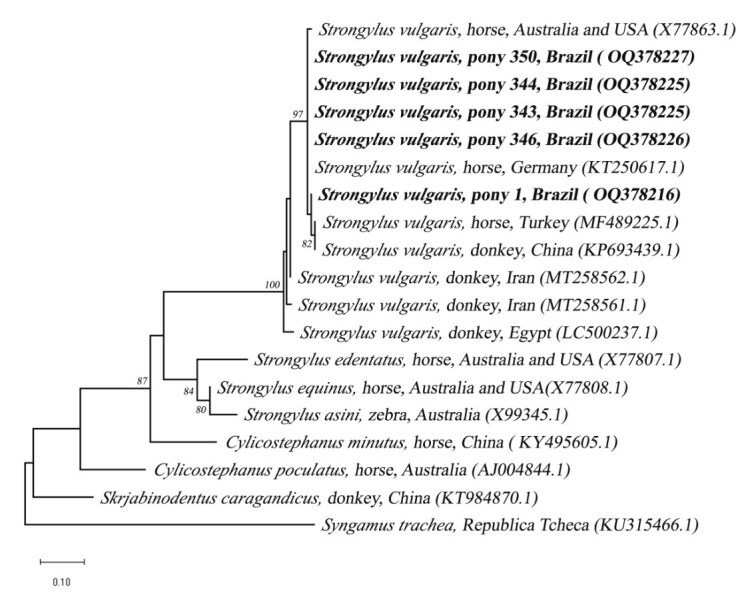
Phylogenetic tree based on the alignment of a 169-bp DNA fragment from the ITS2 region of nematode species, using the maximum likelihood method with the evolutive model TN+F+G4. Sequences from the present study have been highlighted with a black circle. The nucleotide sequence of the *Syngamus trachea* was used as an external group.

Out of the 27 samples, 26 (96.3%) amplified DNA products compatible with the expected size for *S. vulgaris*. After sequencing, it was found that 12 samples presented nucleotide sequences that were good enough for interpretation. From the topography of the phylogenetic tree, it was observed that all the sequences were in the same cluster of sequences of *S. vulgaris* as those from other countries (Figure 1). The degrees of similarity ranged from 99.3 to 100%, in comparison with sequences generated in this study from the reference samples held in GenBank (X77863.1 and KT250617.1).

Out of the 12 fecal samples with confirmed nucleotide sequences from *S. vulgaris*, 10 were from ponies on farm A, one from B, and one from C. Ten of these 12 samples were from females, of which seven were from animals under three years old. These 12 amplified nucleotide sequences from the ITS2 region were deposited in GenBank under the accession numbers OQ378216 to OQ378227. It should be noted that we were unable to find any other sequences representing *S. vulgaris* in ponies anywhere, which thus classifies the present samples as the first of their kind in the world.

## Discussion

In this preliminary study, the frequency of helminths detected in the Brazilian Pony breed was 81.4%. There is not a great variety of scientific data on this breed in the Brazilian literature and, therefore, we made comparisons with other pony breeds in different countries. Frequencies of gastrointestinal helminths lower than that of the present study were reported among ponies in Australia (79.2%) and Israel (36.3%), through the modified McMaster coproparasitological technique, and in India (20.6%) through the Stoll, floatation and sedimentation techniques (Flanagan et al., 2013; Levy et al., 2015; Matto et al., 2015). Only Živković et al. (2021) presented data with prevalence higher than ours, from ponies in Serbia (97.4%). The differences in frequency among the abovementioned parasitological surveys may be related to the distinct geographical locations of the farms, the health management implemented, and the length of time the animals spent on pasture. In addition, the laboratory techniques used for investigating the parasites may also have influenced the parasitological frequencies reported, considering that in the abovementioned studies, a quantitative technique of lower sensitivity (McMaster) than the Mini-FLOTAC technique was used (Ghafar et al., 2021).

The only parasites identified in the fecal samples from these animals of the Brazilian Pony breed were strongylids and *Parascaris* spp. Although the prevalence detected was high in the number of animals sampled, the EPG count was below 500. In correlating the EPG data with the clinical findings, we considered that the level of infection was mild to moderate, by the classification indicated in the guide of the American Association of Equine Practitioners (Nielsen et al., 2019). Like in the present study, EPG counts for strongylids below 500 were reported in most ponies in a study conducted in Israel and in all ponies in Serbia (Levy et al., 2015; Živković et al., 2021). These low estimated EPG values in ponies may be associated with the hardiness of the breed and consequently the resilience of these animals. Further studies for determining the sensitivity of equine breeds to parasite infections are recommended.

Although our results are descriptive, it could be seen from the number of fecal samples used in this study that the highest EPG counts both for strongylids and for *Parascaris* spp. were obtained through the NaCl solution (d = 1.200 g/ml). Moreover, this solution made it possible to diagnose the largest number of positive samples for these parasites. Our greater recovery of strongylid eggs through the NaCl solution was in line with other data from Mini-FLOTAC (Cringoli et al., 2010, 2017). These results demonstrate a tendency to use the NaCl solution in the Mini-FLOTAC technique in parasitological field surveys on samples from ponies. Even so, more studies, including a larger sample panel, need to be performed, since the results of the EPG showed a high standard deviation, so the mean values ​​did not reflect the egg count of all the stools analyzed.

In the macroscopic evaluation, it could be seen that all the ponies had feces of solid consistency with a brown to greenish-brown color. No adult forms or fragments of helminths were recovered during the evaluation. Nonetheless, it needs to be emphasized that the ponies may have presented asymptomatic or sub-clinical parasite infections.

It is known that *Parascaris* spp. is one of the biggest problems regarding the health of young horses (Clayton & Duncan, 1979). These parasites may cause notable alterations in the digestive system, such as intestinal impaction and perforation, which may evolve into severe and fatal cases (Barriga, 1995). Although the great majority of the parasitized pony were male, eggs of *Parascaris* spp. were exclusively detected in the females and mostly in samples from young animals of less than three years of age (one pony was six months old and three were 1 year old). Similar findings regarding *Parascaris* spp. have been reported from parasitological surveys on other breeds of horses in Ethiopia (Chemeda et al., 2016). On the other hand, the frequency of findings of *Parascaris* spp. was greater among males than among females in a study on horses in Germany (Rehbein et al., 2013). Immunity against this nematode develops over the course of the first year of life of horses, through successive infections (Chemeda et al., 2016). It should be noted that the finding from the present study of infection due to this nematode exclusively in females was only from farm A. This was the farm on which the mares were housed together with their foals during lactation. This situation may have contributed to maintaining infection in the mares, given that young animals are more susceptible to infection by these nematodes. However, due to the low sampling number, it was not possible to associate the frequency of positive fecal samples posit with the age group.

The health management procedures that were made available for the ponies, such as the interval between bedding changes, the use of anthelmintics, the drug dose calculations, and the interval between drug administrations and the source of water were generally similar between the stud farms. Coproparasitological diagnoses were not made as a routine procedure on any of the farms. Antiparasitic agents, mostly ivermectin, were supplied at three-month intervals for preventive control of parasite infections. The presence of *S. vulgaris* DNA in the ponies' feces in this study may indicate a possible resistance of this nematode to ivermectin. Due to the long prepatent period of *S. vulgaris* (6 to 7 months), this parasite would disappear if animals were treated every three months with an effective anthelmintic (Barriga, 1995; Carvalho, 2006).

DNA from *S. vulgaris* was identified in 26 fecal samples from ponies. However, it was only possible to confirm nucleotide sequences belonging to *S. vulgaris* in the DNA products from 12 samples. Among these, five sequences that were inserted in the phylogenetic tree (Figure 1) presented base-pair sizes compatible with reference sequences that had previously been deposited in the GenBank. It could be seen that almost all the nucleotide sequences of *S. vulgaris* that were generated from the fecal samples from the Brazilian Pony breed presented high similarity to those coming from horses in Germany, Australia, and the United States (Campbell et al., 1995; Kaspar et al., 2016). The exception was the sequence generated from sample 1 (GenBank OQ378216), which was closer to sequences from fecal material coming from a donkey reared in China and a horse reared in Turkey, as seen in comparisons with material that had been deposited in the GenBank.

Almost all the fecal samples from the ponies in this study (96.3%) were found to be positive for *S. vulgaris*. Lower positivity rates than in the present study, were also obtained through PCR from horses in Denmark (12.1%), and Germany (1.9%), and among horses and donkeys in Iran (8.8% and 9.7%, respectively) (Bracken et al., 2012; Kaspar et al., 2016; Alborzi et al., 2020). The presence of DNA from this parasite in the feces of different equids that were kept in different countries emphasizes the widespread distribution of this nematode. However, the present study is the first coproparasitological survey conducted in Brazil to confirm the presence of *S. vulgaris* in feces from equids using a molecular tool.

The high sensitivity of PCR ended up being fundamental for diagnosing *S. vulgaris*, considering that this nematode was even detected in fecal samples that had been considered negative (based on the EPG count) or presented low counts. Similar findings were reported in diagnosing *S. vulgaris* in equids in Denmark and Iran (Bracken et al., 2012; Alborzi et al., 2020). *S. vulgaris* is considered to be the intestinal nematode species that are highly pathogenic for equids, given the occurrence of thromboses and aneurysms in the mesenteric arteries, due to the migration of its larval forms (Carvalho, 2006). This pathogenicity emphasizes the need for monitoring this parasite through PCR, especially in breeds that have a higher sensitivity to infection. The present study highlights the need for further clinical and laboratory studies, given that little or nothing is known about the pathogenesis of *S. vulgaris* in ponies, as well as the possibility of resistance of this nematode to anthelmintics (i.e., ivermectin) commonly supplied to these animals.
